# Tumor-infiltrating lymphocytes demonstrate potent anti-tumor efficacy and synergize with PD-1 blockade in bladder cancer

**DOI:** 10.1186/s12967-026-07924-6

**Published:** 2026-02-26

**Authors:** Guan-Kai Huang, Xin-Xin Zhang, Lin-Yuan Huang, Hio-Cheng Un, Ren-Xuan Lin, Jun-Xing Chen, Zong-Ren Wang, Ling-Li Long

**Affiliations:** 1 Department of Urology, The First Affiliated Hospital, Sun Yat-Sen University, Guangzhou, 510080 China; 2 Department of Urology, Guangzhou Women and Children’s Medical Center, Guangzhou, 510080 China; 3 Institute of Precision Medicine, The First Affiliated Hospital, Sun Yat-sen University, Guangzhou, Guangzhou, 510080 China; 4 Clinical Trials Unit, The First Affiliated Hospital, Sun Yat-sen University, Guangzhou, 510080 China

**Keywords:** Bladder cancer, Tumor infiltrating lymphocytes, Patient-derived organoids, Single cell sequencing, PD-1 blockade

## Abstract

**Background:**

Bladder cancer (BCa) is one of the most prevalent urological malignancies globally with substantial clinical challenges characterized by high recurrence rates and limited treatment options for advanced disease. Although immune checkpoint inhibitors (ICIs) provide clinical benefit, their efficacy is restricted, with objective response rates of only 20–25%. Adoptive cell therapy (ACT) using tumor-infiltrating lymphocytes (TIL) has shown remarkable success in other solid tumors but remains largely unexplored in BCa. The emergence of patient-derived organoids (PDO) offers a physiologically relevant ex vivo platform for functionally evaluating TIL by preserving tumor heterogeneity.

**Methods:**

TIL were isolated from BCa patient specimens and robustly expanded ex vivo using interleukin-2 (IL-2) and a rapid expansion protocol (REP). Expansion efficiency was quantified, and TIL phenotypes were characterized via flow cytometry. Transcriptional and clonal dynamics were profiled using single-cell RNA sequencing (scRNA-seq) coupled with T-cell receptor (TCR) sequencing. The anti-tumor efficacy of TIL, both as monotherapy and in combination with the PD-1 inhibitor Nivolumab, was assessed against BCa cell lines and autologous PDO through viability (ATP), cytokine release (IFN-γ, ELISA), and apoptosis (caspase 3/7) assays. In vivo validation was performed in a 5637 cell-line-derived xenograft (CDX) mouse model.

**Results:**

TIL were successfully expanded from 33 of 48 BCa samples with high yields of viable cells. Expanded TIL were predominantly CD8 + cytotoxic T lymphocytes. Integrated scRNA and TCR-seq analysis revealed that REP enriched for cytotoxic effector clones and reduced regulatory T-cell populations. TIL mediated potent tumor-killing efficacy against BCa cell lines and autologous PDO in vitro and significantly suppressed tumor growth in vivo. Critically, combining TIL with Nivolumab synergistically enhanced tumor cell death in PDO and resulted in near-complete tumor suppression in the CDX model, significantly outperforming TIL monotherapy.

**Conclusions:**

TIL isolated from bladder cancer patients can be robustly expanded in vitro into a population enriched with CD8⁺ T cells, which exhibited potent anti-tumor activity across both in vitro and in vivo models. Notably, combining TIL with PD-1 blockade significantly enhanced efficacy, establishing TIL-based adoptive cell therapy as a viable immunotherapeutic strategy for bladder cancer and provide a compelling rationale for the clinical translation of TIL combined with Nivolumab.

**Supplementary Information:**

The online version contains supplementary material available at 10.1186/s12967-026-07924-6.

## Introduction

Bladder cancer ranks among the most common malignancies worldwide and is characterized by high recurrence rates and suboptimal treatment outcomes, collectively contributing to a substantial clinical and economic burden. Although surgery serves as a conventional intervention, it is associated with considerable drawbacks and risks. Transurethral Resection of Bladder Tumor (TURBT) is the primary surgical intervention. However, the overall recurrence rate following TURBT remains high, ranging from 50% to 70%. To mitigate recurrence, intravesical Bacillus Calmette–Guérin (BCG) instillation is commonly recommended as an adjuvant immunotherapy to stimulate patient-specific immune responses. However, its efficacy remains limited.Therefore, we underscore the urgent need for more effective immunotherapeutic strategies after surgery.

Tumor-infiltrating lymphocytes (TIL), naturally primed against tumor antigens, offer a compelling approach to harness the immune system for tumor-specific eradication. TIL-based adoptive cell therapy (ACT) leverages ex vivo expansion of autologous TIL followed by reinfusion, enabling precise tumor targeting without chemotherapy-associated toxicity and reduced dependency on complex medical infrastructure. This therapy has demonstrated remarkable clinical efficacy in several solid tumors such as cervical cancer, non-small cell lung cancer (NSCLC), and melanoma [1, 2]. This is most notably exemplified in metastatic melanoma, where durable response rates of 50–70%—with many complete responses lasting over a decade—have paved the way for the first FDA-approved TIL therapy [3]. However, the application of TIL in bladder cancer—despite the tumor’s immunogenic potential—remains in a nascent phase of investigation. Soave et al. has shown that TIL can be successfully isolated from primary bladder tumors via TURBT, and subsequently expanded ex vivo to yield reactive T-cells with anti-tumor activity [4]. Nonetheless, the further functional validation and therapeutic potential of TIL in bladder cancer patients are still underexplored, underscoring the necessity for more profound and systematic research to translate these preliminary findings into viable clinical applications.

To effectively evaluate TIL efficacy against bladder tumors, patient-derived organoids (PDO) represent the optimal model due to their capacity to recapitulate the patient-specific genetic, molecular, and pathophysiological characteristics. Currently, PDO in bladder cancer primarily serve as functional models for predicting responses to cancer drugs such as standard-of-care chemotherapies (cisplatin/gemcitabine), as well as targeted agent screening of antibody-drug conjugates (ADCs) [5, 6]. However, the PDO’ application in functional assays evaluating the efficacy of TIL remains limited, highlighting the need for further exploration.

Given that obtaining sufficient functional TIL through in vitro expansion remains one of the major challenges in bladder cancer TIL therapy [7], we have optimized the culture medium formulation and protocol to successfully enhance the ex vivo expansion of bladder cancer-derived TIL. We comprehensively evaluated TIL therapy through an integrated approach in this study. We first optimized TIL isolation and expansion across clinicopathological subtypes, then characterized phenotypic and clonal evolution of expanded TIL via scRNA-seq and TCR-seq. Using PDO with genomic and histopathological fidelity to parental tumors, we modeled TIL-tumor interactions and enabled physiologically relevant assessment of TIL cytotoxicity. Finally, we evaluated in vitro and in vivo anti-tumor efficacy of expanded TIL, including synergy with programmed death-1 (PD-1) blockade.

Our findings demonstrate robust expansion of CD8 + dominant TIL enriched for cytotoxic clones, significant tumor killing in PDO and xenograft models, and synergistic efficacy when combined with Nivolumab, a PD-1 inhibitor. This synergy overcomes TME-driven immunosuppression, positioning TIL-based ACT as a translatable strategy for bladder cancer.

## Result

### Patient demographics and TIL processing feasibility

**Fig. 1:**
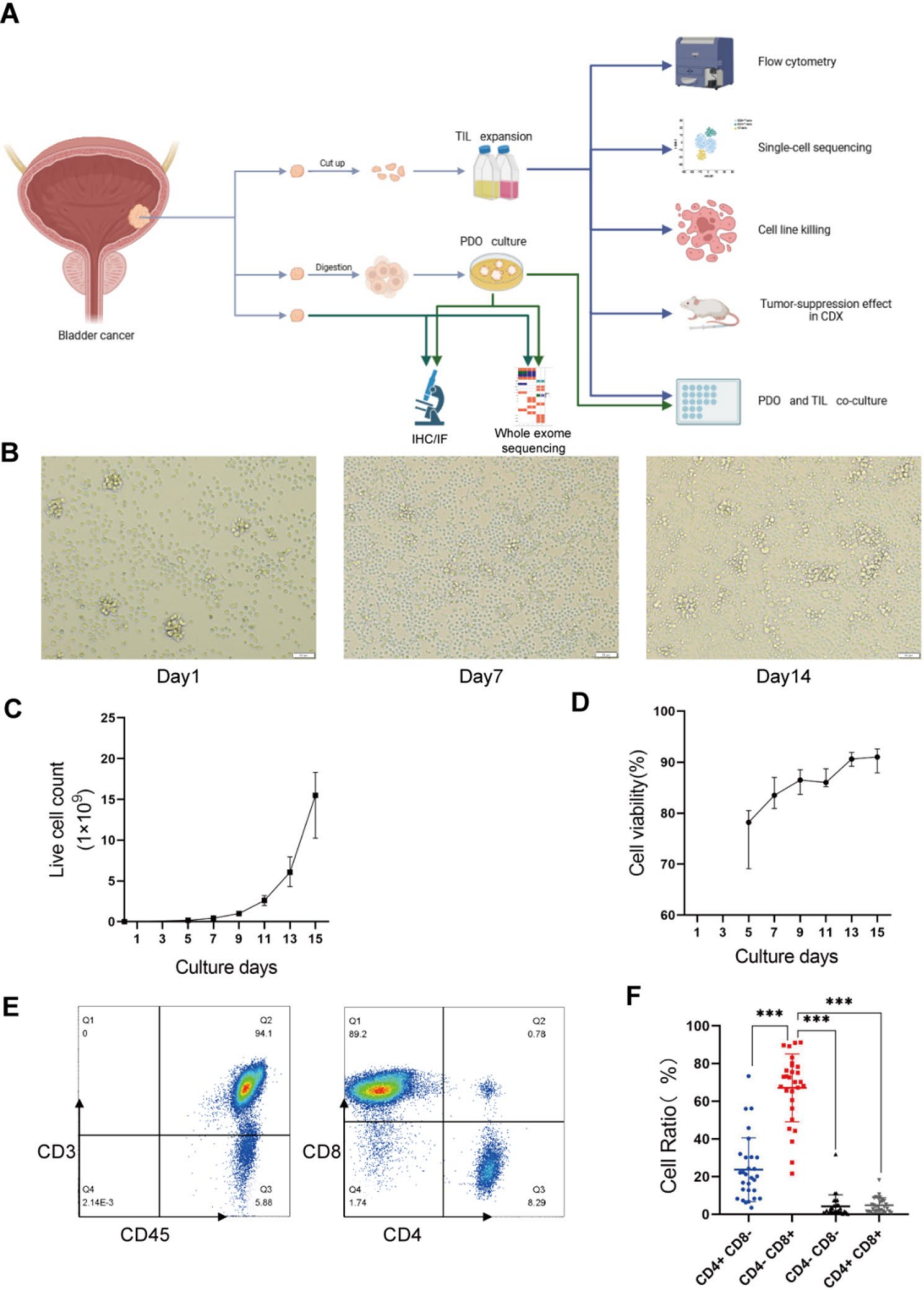
The feasibility and phenotype of TIL from bladder tumor specimens. (**A**) Schematic of the study workflow. TIL represents tumor-infiltrating lymphocyte. PDO represents patient-derived organoid. CDX represents cell-derived xenograft. (**B**) Representative microscopy images illustrating TIL exhibiting cluster-like growth patterns during the initial culture, purification, and rapid expansion protocol (REP) phases. (**C**) The kinetic curve of TIL expansion. Live cell counts over culture days are shown with data presenting as median value ± interquartile range(IQR). (**D**) The TIL viability curve. Cell viability over culture time is presented with data presenting as mean value ± SD (triplicate measurements per TIL sample). (**E**) Representative flow cytometry plots gated on live lymphocytes revealing phenotypes of TIL. (**F**) Quantitative analysis of CD4⁺ and CD8⁺ T cell frequencies within TIL derived from bladder carcinoma specimens (*n* = 29). Data are presented as mean ± SD. Paired t-test. ***P < 0.001

To assess the feasibility of isolating and expanding tumor-infiltrating lymphocytes (TIL) from bladder tumors, we developed an optimized TIL culture system using a refined protocol specifically tailored for specimens obtained via transurethral cystoscopy. The overall workflow of this study is illustrated in Fig. 1A. Fresh tumor specimens were surgically resected from 48 bladder cancer patients. Using high-dose IL-2 (4000 IU/mL), TIL were successfully isolated and expanded from 33 of these 48 specimens (69%). Pathological examination confirmed that these specimens comprised 4 bladder papillary tumor tissues and 29 bladder carcinoma tissues. Detailed sample characteristics, including tumor stage, grade, and patient demographics, are presented in Table 1. There were no statistically significant differences in age, sex, grade of pathology, Tumor stage, or method of sample collection between patients from whom TIL expansion succeeded versus failed (p-value > 0.05; Table 1), indicating the robust and broad applicability of our TIL expansion protocol.

**Table 1:** Clinical characteristics of the included bladder cancer patients

Baseline Characteristics	Participants (*n* = 48)	TIL Expansion Failed (*n* = 15)	TIL Expansion Succeeded (*n* = 33)	Success Rate	*P*-value^#^
Age, years (mean ± SD)	65.65 ± 12.10	66.53 ± 6.47	65.24 ± 14.00	-	0.664*
Sex, (n)					0.700
Males	39	13	26	66.7%	
Females	9	2	7	77.8%	
Grade of pathology, (n)					0.582
Low-grade	11	5	6	54.5%	
High-grade	33	10	23	69.7%	
T stage					0.281
Ta	15	6	9	60.0%	
T1	16	6	10	62.5%	
T2	5	0	5	100.0%	
T3 and above	8	3	5	62.5%	
Papilloma	4	0	4	100.0%	
Method of sample collection					0.742
Transure cystoscopic resection	32	11	21	65.6%	
Radical cystectomy	16	4	12	75.0%	

^#^ P-values compare the distribution of each characteristic between the “TIL Expansion Failed” and “TIL Expansion Succeeded” groups. Categorical variables were analyzed using Pearson’s Chi-square test or Fisher’s exact test, as appropriate

* The p-value of age was compared using an independent two-sample t-test

### Expansion and phenotype of TIL from bladder tumor specimens

To assess the total growth potential of TIL derived from bladder cancer, we monitored the long-term expansion kinetics of TIL cultured from 29 bladder carcinoma tissue samples. Our analysis revealed that IL-2-stimulated TIL consistently formed distinct cell clusters and underwent rapid proliferation during the initial culture phase (Day 1), purification process phase (Day 7), and rapid expansion protocol (REP) phase (Day 14) (Fig. 1B). The final TIL yield exhibited considerable variation across patient samples. After just 15 days of culture, a median yield of 15.48 × 10^9 TIL was obtained from the 29 bladder carcinoma tissues, with a range from 3.06 × 10^9 to 35.31 × 10^9 (Fig. 1C). Critically, all successfully expanded samples yielded at least 100 million TIL, meeting the typical threshold required for adoptive cell therapy in solid tumors [8]. Cell viability, assessed as shown in Fig. 1D, progressively increased during the culture phase, reaching and stabilizing at approximately 90%. This standardized procedure demonstrated reliability and reproducibility, thereby establishing a robust source of tumor-specific T cells for subsequent functional analyses and therapeutic applications.

To characterize the phenotypic profile of the expanded bladder cancer TIL, we evaluated the expression of T-cell differentiation markers following REP. Flow cytometric analysis (Fig. 1E-F) indicated that the majority of expanded TIL were CD8⁺ T cells (67.14% ± 18.01%), while CD4⁺ T cells formed a minority of the population (23.69% ± 16.93%). The residual CD3⁺ TIL falling outside these single-positive gates primarily consisted of a minor population of double-negative (CD4⁻CD8⁻) and double-positive (CD4⁺CD8⁺) cells. This pronounced bias towards CD8⁺ T cells signifies preferential enrichment of cytotoxic T lymphocytes (CTL), thereby enhancing the population’s inherent tumor-killing capacity.

Collectively, these findings confirm the feasibility of generating large quantities of CD8⁺-enriched TIL from diverse bladder cancer specimens under standardized culture conditions. By providing a consistent and scalable platform for the production of cytotoxic lymphocytes, this approach establishes a foundation for the further development and clinical evaluation of TIL-based cellular immunotherapies for bladder cancer.

### scRNA-seq + TCR-seq reveals transcriptome and clonal dynamics of in vitro-expanded bladder cancer TIL

**Fig. 2:**
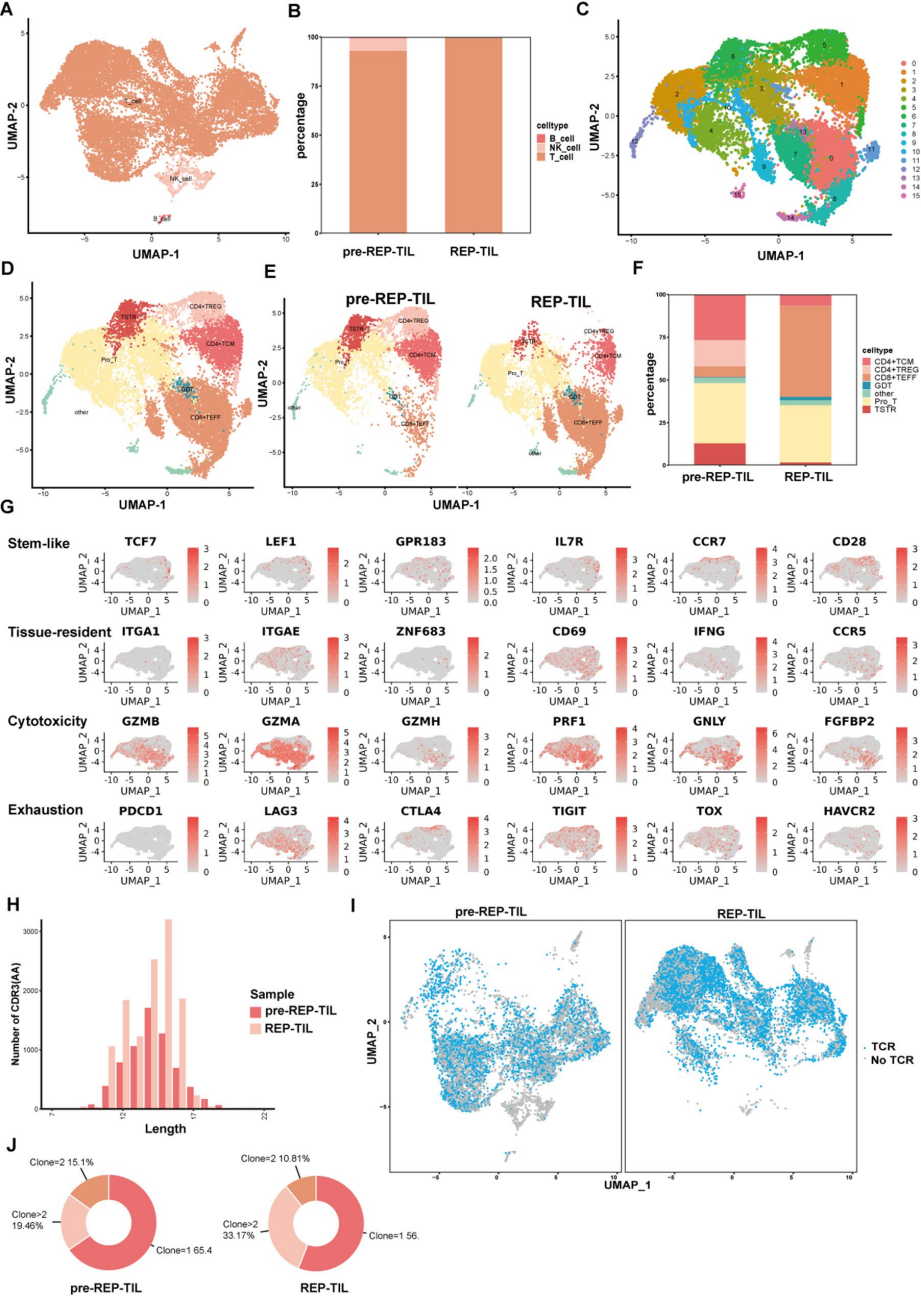
Transcriptome and clonal dynamics of in vitro-expanded bladder cancer TIL. (**A**) UMAP plot of scRNA-seq (*n* = 17,017 cells) colored by major cell lineages. See more details of their canonical markers expressions in Supplementary Fig. 1A. (**B**) Stacked bar plot depicting the proportions of T, B, and NK cells within pre-REP and REP TIL populations. (**C**) UMAP plot of isolated T cells (*n* = 16,444 cells) following dimensionality reduction and clustering, revealing 16 distinct T cell clusters. (**D**) UMAP plot of defined T cell subsets colored by subset identity. See more details of canonical subset markers in Supplementary Fig. 1B. (**E**) UMAP plots comparing T cell subset distribution between pre-REP and REP TIL samples. (**F**) Stacked bar plot depicting the proportions of defined T cell subsets within pre-REP and REP TIL populations. (**G**) Feature plots visualizing scaled expression of representative genes associated with T cell differentiation, cytotoxicity, and immune checkpoints across the T cell UMAP landscape. See more related-genes in Supplementary Fig. 1C. (**H**) Histogram displaying the CDR3 length distribution based on the TCR repertoire. (**I**) UMAP plots comparing clone size distribution within T cells between pre-REP and REP TIL samples. (**J**) Volcano plot identifying proportions between T cell subsets with high-frequency clonotypes (clone > 2) and other T cells (clone ≤ 2)

To further characterize the phenotypic landscape of TIL, we performed paired scRNA-seq and TCR-seq on TIL from matched pre-REP and REP samples. After standard preprocessing, 17,017 high-quality TIL were identified. Besides T cells, minor populations of natural killer (NK) cells and B cells were detected (Fig. 2A; Supplementary Fig. 1A). Proportional analysis revealed these non-T cells were predominantly derived from pre-REP TIL, whereas REP TIL comprised exclusively T cells (Fig. 2B), indicating that long-term in vitro expansion yields purified T-cell populations free from other cellular influences.

From these TIL, 16,444 T cells were analyzed. UMAP dimensionality reduction and clustering identified 16 distinct clusters (Fig. 2C). Based on canonical marker expression (Supplementary Fig. 1B) [9–11], these clusters were categorized into seven T-cell subsets: CD4⁺ TCM, CD4⁺ TREG, CD8⁺ TEFF, Pro_T, TSTR, GDT, and other T cells (Fig. 2D).

Notably, CD8⁺ TEFF cells increased significantly while the Pro_T proportion remained stable in REP TIL, driving an overall expansion of CD8⁺ T cells (Fig. 2E-F). And Cluster 13, characterized by high TRDC expression (encoding TCR δ chain, a γδ T-cell hallmark) [12, 13], was identified as GDT cells capable of MHC-independent antigen recognition. This subset al.so increased post-REP. These results demonstrate robust in vitro TIL proliferation where enhanced CD8⁺ T-cell cytotoxicity and sustained Pro_T retention support expansion.

Conversely, CD4⁺ T cells decreased in REP TIL, particularly the CD4⁺ TREG subset – a regulatory population associated with immune resistance [14]. The TSTR population (Cluster 6), characterized by elevated HSP gene expression (HSPD1, HSPE1) and potentially reflecting cellular stress/dysfunction [9], also showed reduced proportions. These reductions suggest REP-amplified TIL exhibit enhanced antitumor capability suitable for therapy.

Analysis of cytotoxicity and immune checkpoint mRNA profiles in CD8⁺ and CD4⁺ T cells (Fig. 2G; Supplementary Fig. 1C) revealed CD8⁺ T cells express high levels of cytotoxic genes (e.g., PRF1, GNLY) but minimal checkpoint genes (PDCD1, CTLA4). Conversely, a CD4⁺ T-cell subpopulation expressed PDCD1 and CTLA4. These findings reinforce the superior antitumor capability of CD8⁺ TIL, underscoring the importance of prioritizing their expansion for adoptive therapy.

TCR repertoire analysis revealed a normal CDR3 length distribution (Fig. 2H, Supplementary Fig. 1D), indicating TCR stability and typical diversity. Defining high-frequency clones (frequency > 2), we observed their proportion increased post-REP (Fig. 2I-J), confirming clonal expansion with TCR retention. Cells harboring high-frequency clones expressed elevated cytotoxicity genes (NKG7, GZMA), suggesting in vitro expansion selectively amplifies cytotoxic clones, potentially enhancing functionality in subsequent adoptive transfer therapy.

### Expanded TIL demonstrated significant anti-tumor efficacy against bladder cancer in both in vitro and in vivo models

**Fig. 3:**
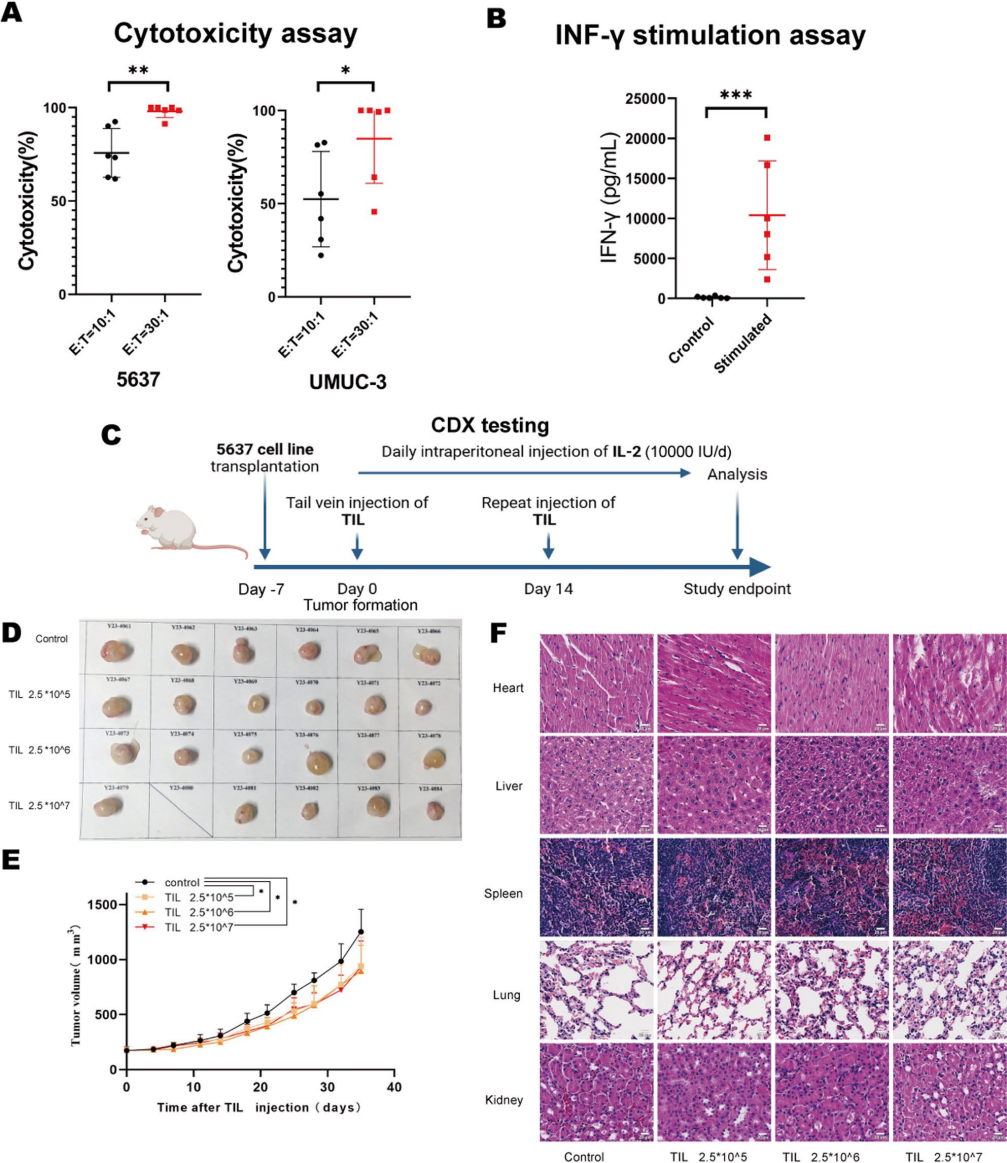
Potent anti-tumor efficacy of TIL against bladder cancer in both in vitro and in vivo models. (**A**) Scatterplot showing tumor-specific cytotoxicity of TIL against bladder cancer cell lines (5637, UM-UC-3) by cytotoxicity assays. Data represents mean ± SD from 6 independent experiments. Paired t-test. *P < 0.05, **P < 0.01. (**B**) IFN-γ production in 5637-TIL co-cultures. IFN-γ levels in co-culture supernatants were measured by ELISA. Data represent mean ± SD (*n* = 6 independent experiments). Paired t-test. ***P < 0.001. (**C**) Experimental scheme for monitoring tumor growth and TIL therapy. (**D**) Xenograft tumor pictures of of indicated 5637 cells subcutaneously injected into NGC mice with different concerntration of TIL (*n* = 6 mice/group). (**E**) Time course of tumor growth in different groups adoptively transferred with different concertrations of human TIL isolated from bladder cancer patients (*n* = 6 mice/group). Linear mixed-effects models with Tukey’s post hoc test. *P < 0.05. (**F**) Histopathological analysis of major organs (heart, liver, lungs, spleen, kidneys) by representative HE-stained sections from NCG mice. Scale bar: 100 μm

To evaluate the anti-tumor efficacy of TIL, we assessed their direct cytotoxicity against human bladder cancer cell lines (5637 and UMUC-3), derived from bladder urothelial carcinoma, using complementary in vitro and in vivo approaches. For in vitro analysis, six distinct TIL samples were co-cultured with target cell lines at effector-to-target (E: T) ratios of 10:1 and 30:1. ATP quantification revealed TIL-mediated cytotoxicity was significantly enhanced at the higher E: T ratio of 30:1, with pronounced tumoricidal activity specifically observed against the 5637 cell line (Fig. 3A). Furthermore, co-culture with 5637 cells stimulated substantially elevated IFN-γ levels in supernatants compared to unstimulated TIL, confirming antigen-driven IFN-γ secretion and its correlation with cytotoxic activity (Fig. 3B).

In vivo anti-tumor activity was evaluated by subcutaneously implanting the 5637 cell line into NCG mice, followed by intravenous (i.v.) TIL infusion via the tail vein (Fig. 3C). Tumors and major organs (heart, liver, spleen, lung, and kidney) were harvested on day 35 (Fig. 3D-F). TIL administration significantly inhibited tumor growth across all TIL-treated groups compared to the control. Notably, the magnitude of tumor suppression was comparable among groups receiving high-dose (2.5 × 10⁷ cells), medium-dose (2.5 × 10⁶ cells), and low-dose (2.5 × 10⁵ cells) of TIL. This lack of dose-dependent efficacy at the administered doses suggests a potential limitation related to the effective homing or intratumoral bioactivity of adoptively transferred TIL.

One mouse in the high-dose group succumbed within 24 h after the second TIL infusion, potentially due to an underlying experimentally induced infection. Hematoxylin and eosin (H&E) staining of major organs from all TIL-treated, tumor-bearing NCG mice (including the succumbed one) revealed no significant histopathological abnormalities, confirming the safty of TIL (Fig. 3F).

### Establishment of bladder cancer PDO and validation of homology with parental tumors

Intend to evaluate the effective of TIL on bladder cancer patient-derived organoids (bPDO), we construct bPDO model according to the protocol above. We firstly performed comprehensive genomic and histopathological homology analyses comparing PDO to their respective parental tumors. This validation encompassed three clinically heterogeneous bladder cancer cases (with distinct grades of pathology or underwent different surgical procedures): A216 (high-grade urothelial carcinoma, radical cystectomy, RC), A261 (high-grade urothelial carcinoma, transurethral resection of bladder tumor, TURBT), and A440 (low-grade urothelial carcinoma, TURBT), representing distinct clinicopathological contexts.

**Fig. 4:**
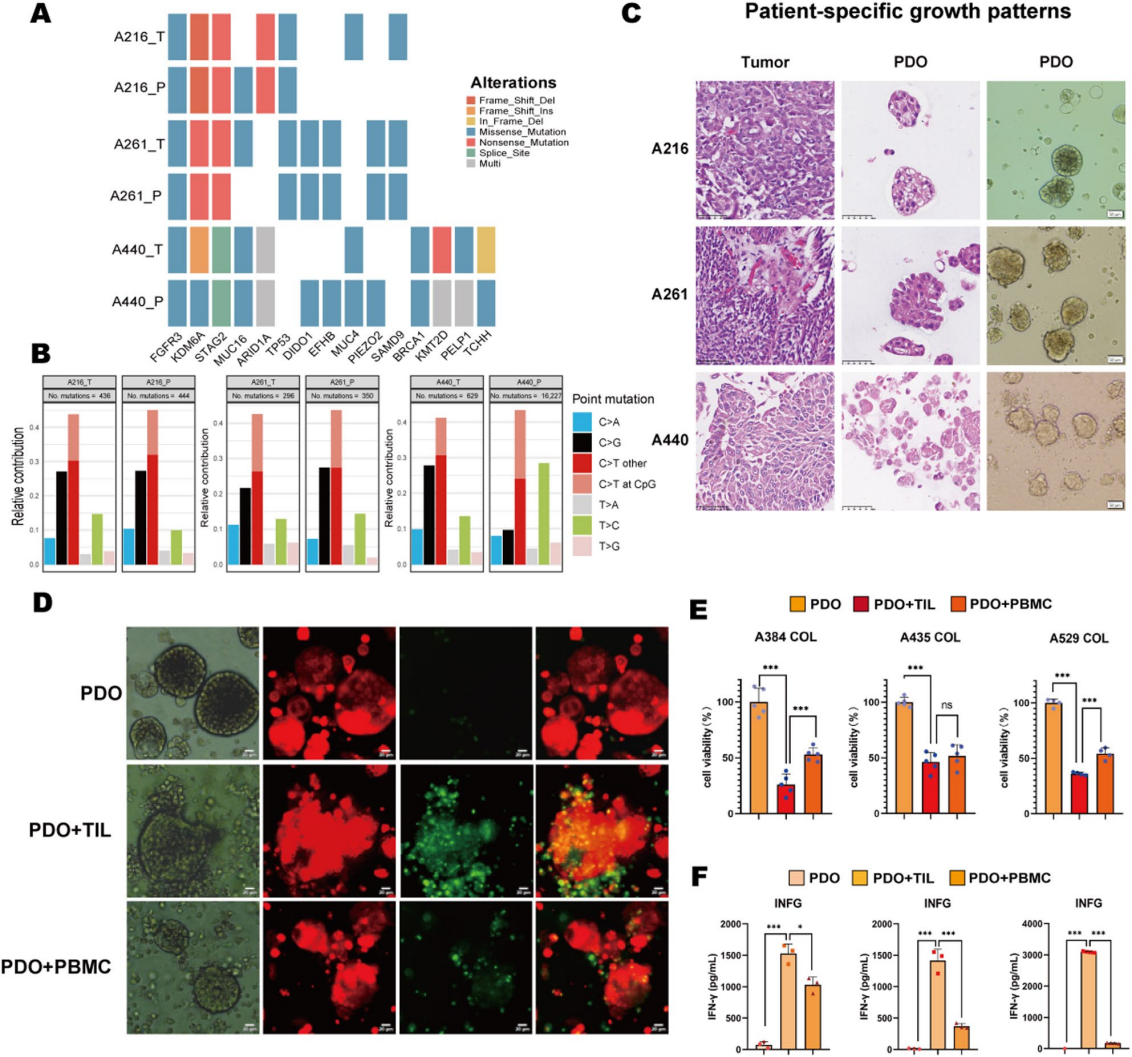
Genomic and histopathological validation of bladder cancer PDO and assessment of their susceptibility to TIL-mediated killing. (**A**) Somatic mutation profile of PDO and matched parental tumors by WES sequencing. (**B**) Single-nucleotide variant (SNV) concordance in tumor-PDO pairs across patients. (**C**) Histopathological fidelity of long-term expanded PDO. Left and middle panels show H&E staining of original tumors and matched PDO, respectively. The right panel displays the bright-field morphology of representative organoids. Scale bar: 50 μm. (**D**) Autologous cytotoxicity assay. Representative fluorescence micrographs of CellTrace FarRed-labeled PDO (red; target cells) from Patient A384 after 24–48 h co-culture. Apoptotic cells are identified by activation of a caspase-3/7 sensor (green). Scale bar: 20 μm. (**E**) Quantitative assessment of TIL-mediated cytotoxicity by ATP-based viability assays. PDO were co-cultured with autologous TIL or PBMC from three patients (A384/A435/A529). Data represents Mean ± SD of 3–5 replicates per group. One-way ANOVA with Tukey’s test. ***P < 0.001, ^ns^*P*>0.05. (**F**) Measurement of IFN-γ level in co-culture supernatants by ELISA assays. Data represents. Mean ± SD from 3 independent experiments. One-way ANOVA with Tukey’s test. ***P < 0.001, *P<0.05

Whole-exome sequencing (WES) of paired parental tumor tissues and derived PDO demonstrated significant concordance in somatic mutational profiles (Fig. 4A). Key shared driver mutations—including those in FGFR3, KMD6A, STAG2, and TP53—were conserved in both PDO and parental tumors, encompassing diverse mutation types such as frameshift deletions/insertions, in-frame deletions, and missense mutations. Furthermore, single-nucleotide variant (SNV) distribution frequencies exhibited substantial overlap between PDO and parental tissues (Fig. 4B). Although a minor discrepancy was noted in PDO derived from patient A440, which showed a marginally higher SNV burden than the matched tumor tissue, the findings collectively confirm that PDO maintain high genomic fidelity to their parental tumors.

We further assessed histopathological and cytomorphological fidelity between long-term cultured bladder cancer PDO and parental tumors using H&E staining and bright-field microscopy (Fig. 4C). Critically, the PDO faithfully recapitulated hallmark pathological features of the original tumors, including nuclear atypia and elevated nuclear-to-cytoplasmic ratios. Importantly, these PDO maintained parental tumor histomorphology across serial passages, ensuring scalability for downstream experimental applications. This robust preservation of intrinsic tumor architecture and molecular characteristics establishes bladder cancer PDO as powerful in vitro models suitable for drug sensitivity profiling and investigations into the tumor-immune microenvironment.

### Evaluation of TIL-mediated tumor killing efficacy in bladder cancer PDO

Leveraging the validated genomic and histopathological fidelity of bladder cancer PDO, we further assessed the efficacy of autologous TIL on their co-derived PDO. For each patient, PDO pre-labeled with CellTrace FarRed (red fluorescence) were subjected to separate autologous co-cultures with their corresponding TIL or peripheral blood mononuclear cells (PBMC) for 24–48 hours, with all experiments independently replicated across the three patients. Apoptosis tracking via caspase-3/7 probes (green fluorescence) revealed significantly enriched apoptotic signals in PDO co-cultured with TIL compared to PBMC control (Fig. 4D), demonstrating TIL’ superior pro-apoptotic activity. Quantitative ATP and IFN-γ ELISA assays collectively demonstrated the potent anti-tumor efficacy of autologous TILs. TIL co-culture induced a significantly more pronounced reduction in PDO viability and a greater elevation in IFN-γ secretion compared to PBMC exposure (Fig. 4E-F). Indeed, one sample (A435) exhibited the reduction in PDO viability mediated by TILs compared to the untreated PDO control, although its effect did not differ significantly from that of PBMC. This observation reflects patient heterogeneity in innate immune cytotoxicity, which may be associated with the younger age of this particular patient. Notably, further analysis using linear mixed-effects models confirmed that, after accounting for inter-patient heterogeneity, the TIL group exhibited significantly superior anti-tumor efficacy compared to both the PBMC and untreated PDO control groups (both *p* < 0.001; Table 2). Importantly, this promising anti-tumor efficacy was attenuated when bPDOs were co-cultured with allogeneic TIL from unrelated patients (Supplementary Fig. 2A-B), confirming stronger antigen-specific immune activation of autologous TIL.

**Table 2:** Linear mixed-effects model analysis of the anti-tumor effects of TIL on bPDOs

LMM-Analysis*	Cell Viability (%)	IFN-γ (pg/mL)
Fixed Effect (Treatment)	F(2, 37.1) = 208.35, *p* = 0.000	F(2, 25.1) = 46.05, *p* = 0.000
Patient Random Effect Variance (σ²)	5.6	35309.1
Residual Variance (σ²)	73.5	262790.6
Conditional R²	0.9	0.74
Marginal R²	0.91	0.77
Fixed Effect Estimated (compare to PDO group)		
PDO+PBMC (Estimate ± SE)	-47.1 ± 3.2	465.4 ± 229.3
PDO+PBMC (t-value, p-value)	t = -14.53, *p* = 0.000	t = 2.03, *p* = 0.053
PDO + TIL (Estimate ± SE)	-63.8 ± 3.2	2095.0 ± 229.3
PDO + TIL (t-value, p-value)	t = -19.68, *p* = 0.000	t = 9.14, *p* = 0.000
Tukey’s Post Hoc Test		
PDO + TIL vs. PDO	*p* < 0.001	*p* < 0.001
PDO + TIL vs. PDO+PBMC	*p* < 0.001	*p* < 0.001
PDO+PBMC vs. PDO	*p* < 0.001	*p* = 0.126
Conditional Marginal Means		
PDO	100	11.8
PDO+PBMC	52.9 ± 2.7	477.2 ± 195.7
PDO + TIL	36.2 ± 2.7	2106.8 ± 195.7

*The model is based on integrated data from three patients (A384, A435, A529), with 3–5 technical replicates per treatment group per patient. Model formula: Response ~ Treatment + (1|Patient). Degrees of freedom were calculated using Satterthwaite’s method. Conditional marginal means incorporate patient-specific random effects. All p-values were adjusted using Tukey’s correction

### TME-driven differential dysregulation of immune checkpoint molecules on TIL

Collectively, these results above demonstrate that TIL are potent agents capable of eliminating tumors. However, the therapeutic efficacy of TIL monotherapy remains limited in clinical settings due to immunosuppressive tumor microenvironments (TME) (e.g., PD-L1-driven T-cell exhaustion) [15, 16]. To overcome this constraint, our subsequent research will focus on the combination strategies integrating TIL with immune checkpoint inhibitors, aiming to amplify antitumor responses through blockade of inhibitory signaling pathways.

**Fig. 5:**
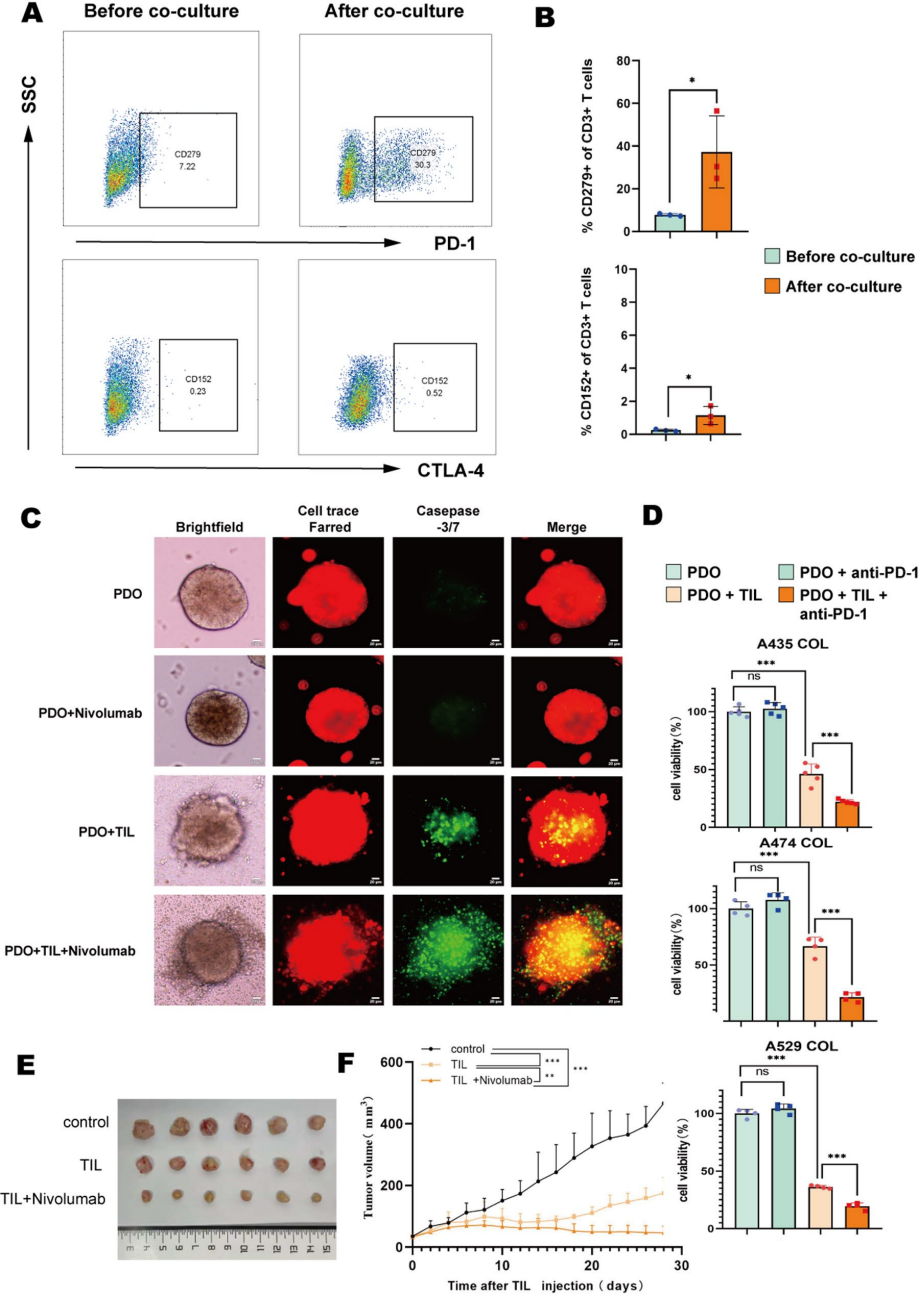
Combining TIL Adoptive Cell Therapy with anti-PD-1 Blockade to Target Checkpoint-Mediated Immune Resistance. (**A**) Representative flow cytometry analysis of PD-1^+^ T cells and CTLA-4^+^ T cells before and after exposure to autologous PDO (patient A435). (**B**) Quantitative summary of PD-1^+^ T cells and CTLA-4^+^ T cells from patient A384, A435, A529. Data are mean ± SD (*n* = 3). Paired t-test. *P < 0.05. (**C**) Representative fluorescence images of PDO (red) under different treatment conditions: TIL, Nivolumab, and TIL combined with Nivolumab or not. Apoptotic cells are identified by caspase-3/7 sensor (green). Scale bar, 20 μm. (**D**) Quantification of the viability of PDO under different treatments: TIL, Nivolumab, and TIL combined with Nivolumab or untreated. Data represents Mean ± SD of 3–5 replicates per group. One-way ANOVA with Tukey’s test. ***P < 0.001, ^ns^*P*>0.05. (**E**) Xenograft tumor pictures of NCG mice with treatments of TIL or TIL combined with Nivolumab, or untreated (*n* = 6 mice/group). (**F**) Tumor growth curve until day 30 post-treatment (*n* = 6 mice/group). Shown as mean ± SD. Linear mixed-effects models with Tukey’s post hoc test. **P < 0.01, ***P < 0.001

To investigate phenotypic dynamics of TIL under simulated bladder cancer TME conditions, we analyzed PD-1 and CTLA-4 expression in TIL before and after autologous PDO co-culture. Flow cytometry revealed that in vitro-expanded TIL without prior PDO exposure exhibited minimal baseline PD-1 and CTLA-4 expression, aligning with prior single-cell RNA sequencing data (Fig. 2G). Importantly, after co-culturing with bPDO, PD-1⁺ T cell proportions increased from 7.77 ± 0.57% to 37.30 ± 16.86% (*p* < 0.05; 4.8-fold increase; *n* = 3), indicating TME-driven PD-1-mediated T-cell exhaustion. Although CTLA-4⁺ T cells also increased (0.24 ± 0.06% to 1.14 ± 0.55%; *p* < 0.05; *n* = 3), absolute levels remained below 2% in all samples (Fig. 5A-B). These findings establish that bladder tumor cells predominantly drive TIL immunosuppression through PD-1 upregulation, with negligible CTLA-4 contribution.

Given these results, we subsequently evaluated the therapeutic potential of combining TIL with anti-PD1 against bladder tumors.

### Synergistic antitumor efficacy of TIL combined with nivolumab

To evaluate the therapeutic potential of combining TIL with anti-PD-1 antibody nivolumab in bladder cancer, autologous TIL and PDO from three patients (A435, A474, A529) were co-cultured for approximately 48 h with or without nivolumab (10 µg/mL). Fluorescence microscopy demonstrated significantly enhanced apoptotic signals in PDO treated with TIL-nivolumab compared to TIL monotherapy or nivolumab alone (Fig. 5C). Quantitative ATP assays corroborated these findings: TIL monotherapy reduced PDO viability to 46.26 ± 8.69% (A435), 66.66 ± 8.11% (A474), and 36.17 ± 1.34% (A529) of controls, while nivolumab alone exerted no direct antitumor effect due to the absence of immune cells in vitro (viability: 102.59 ± 5.25% (A435), 107.96 ± 6.33% (A474), 104.23 ± 3.99% (A529)). Strikingly, the combination therapy exhibited synergistic efficacy, reducing viability to 22.09 ± 1.88% (A435), 21.28 ± 4.01% (A474), and 19.50 ± 2.99% (A529) (Fig. 5D). Further analysis using linear mixed-effects models indicated that the combination therapy group exhibited significantly superior anti-tumor efficacy compared to both the TIL and nivolumab monotherapy groups (both *p* < 0.001; Table 3).

**Table 3:** Linear mixed-effects model analysis of the anti-tumor effects of TIL/antiPD-1 combination therapy on bPDOs

LMM-Analysis*	Cell Viability (%)
Fixed Effect (Treatment)	F(3, 46.0) = 403.22, *p* = 0.000
Patient Random Effect Variance (σ²)	17.8
Residual Variance (σ²)	52.9
Conditional R²	0.95
Marginal R²	0.96
Fixed Effect Estimated (compare to PDO group)	
PDO + TIL (Estimate ± SE)	-50.6 ± 2.9
PDO + TIL (t-value, p-value)	t = -17.73, *p* = 0.000
PDO+anti-PD-1 (Estimate ± SE)	4.7 ± 2.9
PDO+anti-PD-1 (t-value, p-value)	t = 1.66, *p* = 0.103
PDO + TIL+anti-PD-1 (Estimate ± SE)	-79.0 ± 2.9
PDO + TIL+anti-PD-1 (t-value, p-value)	t = -27.68, *p* = 0.000
Tukey’s Post Hoc Test	
PDO + TIL vs. PDO	*p* < 0.001
PDO+anti-PD-1 vs. PDO	*p* = 0.354
PDO + TIL+anti-PD-1 vs. PDO	*p* < 0.001
PDO+anti-PD-1 vs. PDO + TIL	*p* < 0.001
PDO + TIL+anti-PD-1 vs. PDO + TIL	*p* < 0.001
PDO + TIL+anti-PD-1 vs. PDO+anti-PD-1	*p* < 0.001
Conditional Marginal Means	
PDO	100.1
PDO + TIL	49.5 ± 3.2
PDO+anti-PD-1	104.8 ± 3.2
PDO + TIL+anti-PD-1	21.1 ± 3.2

*The model is based on integrated data from three patients (A435, A474, A529), with 4–5 technical replicates per treatment group per patient. Model formula: Response ~ Treatment + (1|Patient). Degrees of freedom were calculated using Satterthwaite’s method. Conditional marginal means incorporate patient-specific random effects. All p-values were adjusted using Tukey’s correction

In vivo validation using a 5637 cell-derived xenograft (CDX) model (*n* = 6 per group) revealed that TIL (2.5 × 10⁶ cells/dose) plus nivolumab (3 mg/kg) significantly reduced tumor volumes versus TIL monotherapy (Fig. 5E). Longitudinal monitoring showed progressive growth in PBS controls (466.75 ± 65.72 mm³), delayed progression with TIL alone (175.41 ± 50.12 mm³), and near-complete suppression with combination therapy (46.42 ± 21.72 mm³; *p* < 0.001 vs. all groups; Fig. 5F).

Integrated analysis revealed patient-specific heterogeneity: A529-derived TIL showed maximal cytotoxicity (36.17% viability reduction) versus moderate efficacy in A474-derived TIL (66.66% reduction). Crucially, nivolumab synergized with TIL across all cases irrespective of baseline activity. This trans-individual synergy underscores the PD-1/PD-L1 axis as a central immunosuppressive mechanism in bladder cancer, indicating PD-1 blockade substantially augments TIL-based therapeutic efficacy.

## Discussion

This study systematically explores the therapeutic potential of tumor-infiltrating lymphocytes (TIL) in bladder cancer. The results show that TIL monotherapy exhibits significant antitumor efficacy both in vitro and in vivo. Notably, the combination therapy with anti-PD-1 (nivolumab) leads to more powerful tumor killing efficacy in both PDO models and animal xenografts. Therefore, we demonstrate the feasibility of ex vivo expansion, intrinsic antitumor activity, and synergistic effects when combined with PD-1 immune checkpoint blockade. Collectively, this study provides, for the first time, a robust experimental foundation for the clinical translation of TIL-based adoptive cell therapy (ACT) in combination with PD-1 blockade for this malignancy.

As a major modality of ACT, TIL therapy leverages tumor-reactive T-cell clones to specifically recognize neoantigens, thereby overcoming limitations of conventional targeted therapies reliant solely on known driver mutations. Although TIL therapy has demonstrated potential for inducing durable remission in advanced solid tumors such as melanoma and cervical cancer, its application in bladder cancer remains nascent. Notably, bladder cancer is regarded as a prototypical “hot tumor”, characterized by a tumor microenvironment (TME) enriched with clonally expanded CD8⁺ T-cell populations, suggesting the presence of a natural antitumor immune foundation. However, two major bottlenecks hinder the clinical application of TIL therapy in bladder cancer: (1) low success rates in primary TIL expansion, attributable partly to reduced tissue viability (e.g., due to thermal damage from electrocautery/laser resection) and microbial contamination risks associated with the bladder’s anatomical exposure to urine; and (2) functional exhaustion of T cells during prolonged ex vivo expansion, which diminishes therapeutic efficacy.

To overcome these obstacles, this present study established optimized protocols for tissue procurement and culture that offer distinct advantages over conventional approaches. Unlike traditional methods involving prolonged cold ischemia, surgical specimens were processed immediately after resection, with cold ischemia time strictly limited to 30 min to better preserve cellular integrity. Moreover, patients received intravesical irrigation of low-dose gentamicin-saline preoperatively to reduce the bacterial burden of the urinary tract, thereby minimizing the risk of microbial contamination and enhancing the success rate of TIL expansion. Furthermore, our approach incorporated CD3/CD28 antibodies with standard IL-2-based culture systems to provide sufficient co-stimulation and activation signals, enhancing both expansion efficiency and functional persistence (Fig. 1). These refinements collectively enabled a 69% overall expansion success rate across 48 fresh bladder cancer specimens with comparable baseline clinicopathological characteristics (Table 1), demonstrating a robust, broadly applicable and clinically relevant methodology for generating functional TIL products.

To precisely evaluate anti-tumor function of TIL, the bladder cancer patient-derived organoids (bPDO) models are successfully established in our study. PDO are three-dimensional ex vivo models that recapitulate the genomic, phenotypic, and histopathological heterogeneity of original patient tumors. Unlike conventional 2D cultures, PDO preserve key aspects of the tumor microenvironment, including diverse cell populations and molecular subtypes, making them physiologically relevant tools for therapeutic evaluation [17, 18]. Prior studies have employed bladder cancer-derived PDO to evaluate standard-of-care chemotherapies such as cisplatin and gemcitabine, as well as targeted agent screening of antibody-drug conjugates (ADCs) such as anti-HER2 therapies [19–21]. Moreover, recent studies about head and neck squamous cell carcinoma (HNSCC) have developed PDO-TIL co-culture models to assess T cell-mediated cytotoxicity and immunotherapy responses and demonstrate the significant advantages of PDO in translational research [22], which is still emerging in bladder cancer. It is unanimously agreed that prior to initiating investigator-initiated trials (IITs) or large-scale GCP-compliant clinical studies, PDO-based systems provide robust, human-relevant data that help bridge the gap between preclinical models and patient responses within a controlled, reproducible, and ethically feasible framework [23–25]. The bPDO in our study exhibits faithfully recapitulate the genomic and histopathological features of parental tumors confirmed by whole exome sequencing (WES), supporting its utility as a broadly applicable functional assay (Fig. 4A-C).

Bladder cancer’s inherent molecular heterogeneity may also affect TIL expansion and therapeutic responsiveness. According to the molecular classification proposed by the Sjödahl group [26], the UroB (immune-activated) subtype exhibits high expression of immune genes such as CD8A, IFNG, and PDCD1, along with dense CD8⁺ T-cell infiltration and high TCR clonality—indicating that this subgroup may be particularly amenable to TIL therapy. In our study, flow cytometry revealed CD8⁺ T cells as the dominant subset after expansion, consistent with prior evidence linking CD8⁺ T-cell abundance to improved survival in bladder cancer patients. Moreover, integrated single-cell RNA and TCR sequencing (scRNA/TCR-seq) revealed that rapid expansion protocols (REP) significantly reshape the TIL repertoire in bladder cancer. Post-expansion, we observed pronounced clonotypic expansion within CD8⁺ effector T cells (TEFF), alongside a reduction in regulatory T cells (TREG), validating the effectiveness of our expansion protocol in enriching for tumor-reactive T cells with enhanced antitumor activity. This functional enrichment was further corroborated by our observation that autologous TILs mediated significantly stronger cytotoxicity against patient-derived organoids compared to allogeneic TILs, underscoring the antigen-specific reactivity of the expanded T-cell population.

Clinical and translational studies, including the IMvigor210 and KEYNOTE-045 trials [27, 28], have demonstrated that PD-1/PD-L1 blockade provides a survival benefit in patients with advanced bladder cancer, yet benefits only a subset of patients (~ 30%). Roh et al. demonstrated in melanoma that high TCR clonality were positively correlated with PD-1 treatment response [29], underscoring the close link between TEFF clonotypic expansion and PD-1 therapeutic efficacy. In our study, co-culture of expanded TIL with autologous PDO induced a substantial upregulation of PD-1 on TIL, indicating TME-driven immune exhaustion primarily via the PD-1/PD-L1 axis. As important roles of immune homeostasis maintaining, pathological overexpression of the PD-1/PD-L1 axis within tumor microenvironment imposes an aberrant “braking” on T-cell-mediated anti-tumor responses, representing a dominant contributor of treatment failures. Therefore, we combined TIL with nivolumab, the PD-1 blockade, to enhance the anti-tumor activity. Our study confirmed in PDO and CDX models that TIL combined with nivolumab yielded significant synergistic effects—where TIL provide a diverse repertoire of tumor-reactive clones (including neoantigen-specific ones) while PD-1 blockade reverses exhaustion, together enhancing the depth of antitumor immunity. These findings indicate that the TIL combined with anti-PD1 treatment against bladder cancer is not an isolated incident and a potential human application should be considered.

Despite these encouraging findings, several limitations must be acknowledged: (i) limited sample diversity—all tumors were sourced from a single center, and more samples from multiple centers will be collected subsequently; (ii) discordant culture success—heterogeneous tissue requirements resulted in most samples yielding either PDO or TIL but not both, limiting paired analyses; (iii) the use of immunodeficient models (CDX and PDO) introduces a specific analytical constraint. Within these systems, the observed synergy between TILs and Nivolumab is primarily attributed to the reversal of exhaustion within the adoptively transferred TIL population itself. This framework precludes the evaluation of how PD-1 blockade might concurrently modulate broader, clinically critical components of the tumor immune microenvironment—such as dendritic cell antigen presentation and macrophage polarization. Consequently, by failing to capture these potential secondary immunostimulatory effects, our current models may underestimate the full therapeutic potential of the TIL and Nivolumab combination. These caveats warrant further investigation.

To address these gaps and refine the translational roadmap, future studies should prioritize models that recapitulate human immune complexity. This includes employing humanized mouse models reconstituted with autologous immune components to study TIL therapy within a more physiological TME. Furthermore, spatial transcriptomic and multiplex imaging analyses of treated tumors could elucidate the cellular neighborhoods and signaling networks, moving beyond bulk measurements to a spatially resolved understanding of immune-tumor interactions.

To the best of our knowledge, this study is the first to provide compelling evidence supporting the clinical translation of TIL combined with anti-PD1 therapy in bladder cancer. By refining expansion protocols and elucidating synergistic mechanisms with PD-1 blockade, it lays the groundwork for future innovations. Overcoming key barriers—such as microbial contamination control and T-cell exhaustion—while deepening insights into clone–antigen interactions and multi-target therapeutic strategies, will be essential. Incorporating molecular subtyping and neoantigen landscapes to guide TCR-T design, and advancing into phase I trials with patient selection enriched for PD-1 resistance or high PD-L1 expression, may ultimately enable the leap from “basic research” to “precision immunotherapy” for bladder cancer.

## Methods

### Clinical specimen acquisition and processing

This study enrolled 48 bladder cancer patients admitted to the Department of Urology, The First Affiliated Hospital, Sun Yat-sen University, between 2022 and 2024. All tumor samples were obtained via transurethral resection of bladder tumor (TURBT) or radical cystectomy (RC). Postoperative pathological reports were collected to verify diagnoses. Clinical data, including age, gender, TNM stage, and histological grade, were systematically retrieved and archived from the hospital’s medical record system. Immediately after resection, tumor tissues were placed in 4 °C Tissue Storage Solution (MACS, Miltenyi Biotec 130-100-008) and processed into three aliquots for: (1) expansion culture of tumor-infiltrating lymphocytes (TIL); (2) establishment of patient-derived tumor organoid (PDO) models; and (3) snap-freezing in liquid nitrogen followed by long-term storage at -80 °C. Peripheral blood mononuclear cells (PBMC) were isolated from patient peripheral blood samples using Ficoll-Paque density gradient centrifugation, resuspended in serum-free cell freezing medium (NCM Biotech C40100), and cryopreserved at -80 °C.

### Cell lines and cell culture

The human bladder cancer cell lines 5637 and UM-UC-3, used in this study, were procured from Procell Life Science & Technology Co., Ltd. All cell lines were authenticated by short tandem repeat (STR) profiling and routinely screened for mycoplasma and chlamydia contamination to ensure experimental integrity. UM-UC-3 cells were maintained in MEM medium, while 5637 cells were cultured in RPMI-1640 medium. Complete growth medium was prepared by supplementing the base medium with 10% fetal bovine serum and 1% penicillin-streptomycin. Cells were incubated at 37 °C in a humidified atmosphere with 5% CO_2_. Regular maintenance included changing water pans and sterilizing the incubator to ensure optimal culture conditions.

### in vitro expansion of TIL

TIL were isolated and expanded ex vivo using a tumor fragment culture technique. A previously optimized protocol for cervical cancer TIL expansion [30] was adapted and modified to accommodate the unique growth characteristics of bladder cancer TIL.

#### Tissue processing and primary culture

Fresh bladder cancer tissues from surgical resections were dissected into 1–2 mm³ fragments. Under strict aseptic conditions, fragments were washed with RPMI-1640 medium to remove blood components and necrotic tissue. Washed fragments were distributed into 24-well plates (1–2 fragments per well). TIL expansion medium, consisting of X-VIVO 15 medium (Lonza 04-418Q), recombinant human interleukin-2 (IL-2, Bio-Techne 202-IL-500, final concentration 4000 IU/mL), and 1% penicillin-streptomycin, were added. Cultures were maintained in a humidified incubator at 37 °C with 5% CO_2_ for 7–14 days. Medium was replaced every 2–3 days, and TIL growth was monitored microscopically.

#### TIL purification and cryopreservation

When TIL were observed migrating from fragments and proliferating as suspended single cells, culture supernatants containing TIL were collected, and residual tissue fragments were removed using a 100 μm cell strainer. TIL were counted every other day. Upon reaching a cell count of ≥ 10 × 10⁶, TIL were harvested for purification and cryopreservation. Purification was performed using Ficoll-Paque density gradient centrifugation to remove dead cells and debris. Cell viability was assessed by trypan blue exclusion, confirming > 85% viability. Purified TIL were resuspended in serum-free cryopreservation medium at a density of ≥ 5 × 10⁶ cells per cryovial, gradually cooled to -80 °C, and subsequently transferred to liquid nitrogen for long-term storage (-196 °C).

#### Rapid expansion protocol (REP)

To generate large numbers of highly active TIL, a rapid expansion protocol was employed. Briefly, cryopreserved TIL were rapidly thawed in a 37 °C water bath (1–2 min), diluted with pre-warmed X-VIVO 15 medium, and centrifuged (1500 rpm, 5 min) to remove DMSO. TIL were stimulated with ImmunoCult™ Human CD3/CD28/CD2 T Cell Activator (Stemcell 10970) and co-cultured with irradiated feeder PBMC to provide co-stimulation. Activated TIL were seeded in T75 flasks at an initial density of 0.5 × 10⁶ cells/mL in TIL expansion medium supplemented with 6000 IU/mL IL-2. Cultures were maintained for 7–14 days, with cell counts and viability assessments performed every other day using trypan blue exclusion.

### Bladder cancer tumor organoid culture

#### Primary culture

(1) Tissue Acquisition and Processing: Fresh bladder cancer tissues were collected in 4 °C MACS Tissue Storage Solution and processed within 24 h; (2) Washing and Dissection: Tissues were washed three times with PBS, and necrotic/fibrous components were removed. Tissues were minced into 1–3 mm³ pieces; (3) Tissue Dissociation: Minced tissues were enzymatically digested with Collagenase IV (0.5 mg/mL) at 37 °C with agitation for 30–60 min (duration adjusted based on tissue consistency). Digestion was quenched with double volume of FBS-containing medium. The cell suspension was centrifuged (2000 rpm, 5 min). Mechanical dissociation was performed by vigorous pipetting or filtration through a 70 μm cell strainer to obtain single cells/small clusters, followed by centrifugation (2000 rpm, 5 min); (4) Matrigel Embedding: The cell pellet was resuspended in a 1:1 mixture of ice-cold Matrigel and Bladder Cancer Organoid Complete Medium; (5) Seeding: 30 µL droplets of the cell-Matrigel suspension were pipetted onto pre-warmed 48-well plates and polymerized at 37 °C for 30 min; (6) Medium Overlay: Each well was overlaid with 200 µL of Bladder Cancer Organoid Complete Medium.

Bladder Cancer Organoid Complete Medium consisted of: Advanced DMEM/F12, HEPES (10 mM), GlutaMAX™ Supplement (1×), recombinant human EGF (50 ng/mL), recombinant human FGF10 (100 ng/mL), recombinant human FGF2 (12.5 ng/mL), FGF7 (25 ng/mL), A83-01 (5 µM), recombinant human R-spondin-1 (500 ng/mL), Y-27,632 (10 µM), nicotinamide (10 mM), N-acetyl-L-cysteine (1.25 mM), B-27 Supplement (1×), and recombinant human Noggin (100 ng/mL). Prepared medium was stored at 4 °C and used within two weeks.

#### Culture conditions

Plates were maintained at 37 °C, 5% CO₂. Medium was replaced every 2–3 days. Organoid formation typically occurred within 7–14 days, characterized by cystic or glandular structures.

#### Organoid passaging

Organoids were passaged every 7–10 days or when density reached ~ 70% confluence or central necrosis appeared. (1) Digestion and Harvesting: Medium was removed, and organoids were washed twice with ice-cold PBS. Organoids were collected by disrupting the Matrigel. (2) Enzymatic Digestion: Organoids were dissociated using TrypLE Express (37 °C, 5–10 min), optionally aided by mechanical pipetting to break into small clusters. (3) Re-seeding: Cells/clusters were diluted at a 1:2 to 1:6 split ratio, re-embedded in fresh Matrigel/medium mixture, and cultured as for primary culture. (4) Optimization: Passaging frequency was adjusted based on growth rate. Organoids were used within 15 passages to maintain genomic stability. Organoids were cryopreserved using commercial cryopreservation medium (CryoStor CS10) following standard cell freezing protocols.

### Cytotoxicity assay of TIL against bladder cancer cell lines and organoids

Human bladder cancer cell lines 5637 or UM-UC-3 were used as target cells. Expanded TIL were resuspended in TIL medium containing IL-2 and added to target cell wells as follows: Experimental Groups: ① Effector: Target (E: T) ratio 10:1: 2 × 10⁵ TIL/well (final volume 100 µL: 50 µL target cells + 50 µL TIL). ② E: T ratio 30:1: 6 × 10⁵ TIL/well (final volume 100 µL). Control Groups: ① Target cell control: Target cells only (50 µL target cells + 50 µL TIL medium). ② TIL control: TIL only (50 µL cell line medium + 50 µL TIL). ③ Medium blank control: Medium only (50 µL cell line medium + 50 µL TIL medium). Co-cultures were maintained for 24 h at 37 °C, 5% CO₂. After co-culture, cell viability was quantified by measuring ATP levels using the CellTiter-Glo^®^ 2.0 Assay. To account for background ATP from TIL, corrected luminescence for experimental wells (C_corrected) was calculated as: C_corrected = C_experimental - C_TIL control. Specific killing percentage (K) was calculated as: K (%) = (1 - C_corrected / C_target control) × 100. IFN-γ levels in the supernatant from TIL co-cultured with 5637 cells at an E: T ratio of 30:1 were measured using the Human IFN-gamma Valukine ELISA Kit according to the manufacturer’s instructions. All experiments were performed in triplicate. Data are presented as mean ± standard deviation.

To validate TIL cytotoxicity against patient-derived bladder cancer organoids, organoids were harvested and partially dissociated into single cells for accurate counting to establish an E: T ratio of 10:1. Organoids were resuspended in IL-2-free Bladder Cancer Organoid Complete Medium and co-cultured with autologous TIL derived from the same patient’s tumor, under conditions similar to the cell line cytotoxicity assay. For visualization, organoids were pre-stained with 1 µM CellTrace Far Red dye (Invitrogen #C34572). During co-culture, a Caspase 3/7 Green Detection Reagent (Invitrogen #C10723, 1:2000 dilution) was added to visualize apoptosis. Microscopic images were captured after 24–48 h of co-culture to document morphological changes. Supernatants and cells were collected for further analysis. IFN-γ secretion in the supernatant was quantified by ELISA to assess TIL immune activation. PBMC from the same patient served as a positive control to compare killing efficacy. Post-co-culture, cell viability was quantified using the CellTiter-Glo^®^ 3D Cell Viability Assay to measure ATP levels and calculate cell survival rate. For experiments exploring the combined effect of TIL and the PD-1 inhibitor Nivolumab, an additional group was included with Nivolumab at a final concentration of 20 µg/mL in the co-culture system [31–33]. Experiments were independently repeated 3–5 times. Data are presented as mean ± standard deviation.

### Whole-exome sequencing (WES) and data analysis

WES was performed using the Agilent SureSelect target enrichment system to capture the human exome, followed by high-throughput sequencing on the Illumina NovaSeq 6000 platform. Genomic DNA was extracted from bladder cancer organoids, matched tumor tissues, and peripheral blood samples using the AllPrep DNA/RNA Mini Kit according to the manufacturer’s instructions. Libraries were prepared using the Agilent SureSelect Human All Exon V6 Kit (Agilent Technologies, CA, USA) following the manufacturer’s protocol, involving DNA fragmentation, end repair/A-tailing, adapter ligation, hybridization capture with biotinylated probes, target enrichment using streptavidin beads, and PCR amplification of captured libraries. Sequencing was performed on the Illumina NovaSeq 6000 platform (2 × 150 bp paired-end reads). Peripheral blood samples were sequenced to a depth of 100× (~ 12 Gb/sample), while bladder cancer organoid and tumor tissue samples were sequenced to 200× (~ 24 Gb/sample). Raw sequencing reads were processed using Fastp (v0.19.3) to remove adapters and low-quality bases. Clean reads were aligned to the human reference genome (UCSC hg19) using BWA-MEM (v0.7.15). Post-alignment processing, including duplicate marking, base quality score recalibration, and indel realignment, was performed according to GATK best practices.

#### Variant calling and analysis

Somatic single nucleotide variants (SNVs) and insertions/deletions (INDELs) were identified using GATK Mutect2 (v4.0.4.0) by comparing tumor/organoid samples against matched peripheral blood (germline control). Variants with allele frequency < 0.05 or supported by fewer than 3 reads were filtered out. Annotation was performed using Annovar, integrating information from COSMIC and dbSNP databases. Mutations were associated with known cancer-related genes, functional impact, and clinical data.

#### Copy number variation (CNV) analysis

CNVs were detected using Facets software (v0.16.0).

#### Tumor mutational burden (TMB) calculation

TMB was calculated as the total number of non-synonymous mutations in the protein-coding region divided by the total exonic panel length, reported as mutations per megabase (mutations/Mb).

### Single-cell RNA sequencing (scRNA-seq) and data analysis

Pre-REP and REP TIL from the same patient (ID: A352, diagnosed with muscle-invasive high-grade urothelial carcinoma post-radical cystectomy) were subjected to sequencing. Approximately 12,000 cells per sample were resuspended in 0.04% BSA-PBS. Single-cell libraries were prepared using the Chromium™ Single Cell 5’ platform (10x Genomics) based on GemCode microfluidics technology. Cells, barcoded gel beads, and master mix were co-encapsulated into Gel Bead-In-EMulsions (GEMs). Cell suspension concentration was optimized to 700–1200 cells/µL to maximize single-cell capture efficiency (~ 90–99% empty GEMs). The mRNA libraries were sequenced on an Illumina NovaSeq6000 S1 flow cell with read configuration: Read 1 (26 bp), i7 Index (8 bp), i5 Index (0 bp), Read 2 (91 bp). Sequencing depth was 50,000 read pairs per cell. And the TCR libraries were sequenced on an Illumina HiSeq2500 in Rapid Run mode with read configuration: Read 1 (150 bp), i7 Index (8 bp), i5 Index (0 bp), Read 2 (150 bp). Sequencing depth was 5,000 read pairs per cell.

Raw sequencing data were processed using Cell Ranger (v.3.1) software and aligned to the human GRCh38 reference genome. Integrated analysis of scRNA-seq and TCR-seq data was performed using Seurat (v.4.3.2) to phenotype pre-REP and REP TIL clones. To ensure the retention of high-quality cells and reduce technical noise, the following QC standard was applied: first, cells with either (1) ≥ 25% mitochondrial gene counts (2) > 25,000 or < 500 UMIs (3) < 200 detected genes were excluded; second, low-frequency genes (expressed in < 3 cells) were removed.

#### Dimensionality reduction and clustering

Unsupervised clustering was performed using Seurat. The filtered expression matrix was normalized using Seurat *NormalizeData* function with default parameters, and the top 2000 highly variable genes were identified for principal component analysis (PCA). The optimal number of principal components was based on criteria established through elbow and Jackstraw plots. Batch effects between samples were corrected using the Harmony algorithm [34]. We identified clusters using Seurat FindClusters function (Louvain clustering, default resolution = 1.0), and Uniform Manifold Approximation and Projection (UMAP) was used for non-linear dimensionality reduction and visualization (using default RunUMAP parameters). Cell clusters were annotated based on canonical marker expression, differentially expressed genes (DEGs), cluster relationships, and TCR repertoire clonality.

#### Differential expression analysis

Differentially expressed genes (DEGs) were identified across clusters through the Wilcoxon rank sum test using the *FindMarkers* function (logfc.threshold = 0.25, min.pct = 0.25), with p-values adjusted using the Bonferroni correction. Adjusted p-values < 0.001 were considered significant. A minimum p-value threshold of e⁻³⁰⁰ was set for visualization purposes due to extremely low p-values.

#### TCR data integration and clonotype definition

TCR-seq data were processed with the scRepertoire package (v2.3.0). Raw TCR sequences were first filtered to retain only productive sequences. Clonotypes were defined using the “CTstrict” criterion. Clone frequency was calculated and integrated into the Seurat object using the *combineExpression* function with default parameters.

### Flow cytometry

In vitro expanded TIL or co-cultured cells were harvested, centrifuged (1500 rpm, 5 min), and resuspended in PBS containing 1% FBS. Cell concentration and viability (> 85%) were determined using a hemocytometer and trypan blue staining. Cell density was adjusted to 1 × 10⁶ cells/mL for staining. Aliquots of 1 × 10⁶ cells were incubated with 1 µL HU Fc Block Pure to block Fc receptors. Cells were then stained with a pre-titrated antibody panel targeting surface markers (e.g., CD3, CD4, CD8, CTLA-4, PD-1; concentrations used as per manufacturer’s recommendations) for 15 min at room temperature in the dark. Data were acquired on a CytoFLEX flow cytometer, collecting at least 100,000 lymphocyte-gated events per sample. Data analysis was performed using FlowJo software. Debris was excluded by gating on forward and side scatter (FSC/SSC). Spectral overlap was compensated using single-stain controls and fluorescence minus one (FMO) controls were used for gating strategy validation. Cell subset frequencies were quantified using Boolean gating strategies.

### Hematoxylin and eosin (H&E) and immunohistochemistry (IHC) staining

#### H&E staining

Formalin-fixed, paraffin-embedded (FFPE) tissue blocks were sectioned at 4 μm thickness. Sections were baked at 65 °C for 2 h, deparaffinized in three changes of xylene (10 min each), and rehydrated through a graded ethanol series (absolute, 95%, 75%; 2 min each) to distilled water. All subsequent steps involving organic solvents were performed in a fume hood. Sections were stained with hematoxylin for 10 min, rinsed gently under running tap water for 1 min, differentiated in 1% acid alcohol for 5 s, rinsed again for 1 min, blued in bluing solution for 30 s, and rinsed thoroughly for 5 min. This was followed by counterstaining with eosin for 2 min and a brief rinse (3 s). Sections were dehydrated through graded alcohols, cleared in xylene (three changes, 3 min each), air-dried, and mounted with neutral resin.

#### IHC staining

Deparaffinization and rehydration were performed as described for H&E staining. Antigen retrieval was performed by incubating sections in citrate-based antigen retrieval buffer under high pressure (2 min after reaching pressure). After cooling to room temperature, sections were washed three times in PBST (PBS with 0.1% Tween-20, 5 min each). Endogenous peroxidase activity was blocked by incubation in 3% hydrogen peroxide for 10 min, followed by three PBST washes (5 min each). Tissue sections were circled with a hydrophobic pen, blocked with 5% BSA for 30 min, and incubated overnight at 4 °C with 100 µL of primary antibody at the optimized dilution. The following day, sections were washed three times with PBST (5 min each) and incubated with 100 µL of horseradish peroxidase-conjugated secondary antibody for 30 min at room temperature. After three additional PBST washes, color development was performed using DAB chromogen; the reaction was stopped by rinsing with tap water once optimal staining intensity was observed. Sections were counterstained with hematoxylin, differentiated, blued, rinsed, dehydrated, cleared, and mounted as described for H&E staining.

### Animal experiments

All animal procedures were approved by the Sun Yat-sen University Animal Ethics Committee. To mitigate interference from the mouse immune system, 4-6-week-old immunodeficient NOD.Cg-Prkdcscid Il2rgtm1Wjl/SzJ (NCG) mice were purchased from GemPharmatech Co., Ltd. (Guangdong, China).

#### CDX model and TIL efficacy

A subcutaneous cell line-derived xenograft (CDX) model was established by inoculating 5637 human bladder cancer cells into the left flank of NCG mice. On the day of inoculation, cells were harvested, resuspended in PBS, counted, and adjusted to 3 × 10⁷ cells/mL. Mice were randomly divided into four groups (*n* = 6/group): a control group (vehicle), and three experimental groups receiving low- (2.5 × 10⁵ cells/dose), medium- (2.5 × 10⁶ cells/dose), or high-dose (2.5 × 10⁷ cells/dose) TIL (from patient A215). All mice received a subcutaneous injection of 100 µL 5637 cell suspension. When tumors reached a palpable size (~ 100 mm³), TIL or vehicle were administered via tail vein injection on day 1 (D1) and D8 (total 2 doses). IL-2 (10,000 IU/mouse/day) was administered intraperitoneally from D1-D5 and D8-D12. Tumor size and mouse body weight were measured every other day. Upon observing significant intergroup differences, mice were anesthetized and euthanized by cervical dislocation. Subcutaneous tumors and major organs (heart, liver, spleen, lung, kidney) were harvested, measured with calipers, weighed, and photographed. Parts of the tumors and organs were fixed, paraffin-embedded, sectioned for H&E and IHC staining. Other tumor parts were stored in 4 °C MACS Tissue Storage Solution for subsequent single-cell isolation and analysis.

#### TIL combined with PD-1 inhibitor

The 5637 subcutaneous xenograft model was established as above. Mice were divided into three groups: control (vehicle), TIL alone (medium dose, as above), and TIL (medium dose) combined with Nivolumab (3 mg/kg, tail vein injection, concurrent with TIL on D1 and D8). All groups received IL-2 as described. Mice were euthanized when significant differences were observed. This experiment utilized TIL from patient A352.

### Statistical analysis

All statistical analyses were performed using GraphPad Prism 10 or R software (v4.3.2). The R packages and their versions are detailed in the Methods section. For continuous variables, Normality of data distribution was assessed using the Shapiro-Wilk test, and homogeneity of variances was verified using Levene’s test or Bartlett’s test. Parametric tests (Student’s t-test for two groups, one-way or two-way ANOVA for multiple groups with Tukey’s post-hoc test) were applied when these two assumptions were met; otherwise, equivalent non-parametric tests (Mann‑Whitney U test for two groups, Kruskal‑Wallis test for multiple groups with Dunn’s post-hoc test) were used. Categorical variables were compared using Pearson’s chi‑square test or Fisher’s exact test, as appropriate. To account for inter‑patient variability in PDO co‑culture assays with multiple treatments per patient, as well as longitudinal data of tumor growth curves in mice, linear mixed‑effects models (LMMs) were fitted using the lmer function (lme4 package, v1.1.37) with Tukey’s post-hoc test. In these models, ‘Treatment’ was included as a fixed effect and ‘Patient ID’ or ‘Animal ID’ as a random effect. Significance of fixed effects was assessed using Satterthwaite’s approximation for degrees of freedom. Detailed LMM outputs (variance components, effect estimates, and adjusted p‑values) are provided in Tables 2 and 3. All tests were two‑sided and all reported p‑values for multiple comparisons were adjusted values. Significance levels are denoted as follows: *p < 0.05; **p < 0.01; ***p < 0.001; ns (not significant), *p* > 0.05.

## Supplementary Information

Below is the link to the electronic supplementary material.

Supplementary Material 1

## Data Availability

The datasets supporting the conclusions of this article are included within the supplementary file.
