# Supplementary material for: Tumor-infiltrating lymphocytes demonstrate potent anti-tumor efficacy and synergize with PD-1 blockade in bladder cancer
Source: J Transl Med. 2026 Feb 26;24:455. doi: 10.1186/s12967-026-07924-6 (PMC13041153; doi:10.1186/s12967-026-07924-6)
Supplement: Supplementary file 1 — Supplementary Material 1 [file 12967_2026_7924_MOESM1_ESM.docx]

**Supplementary Figures**


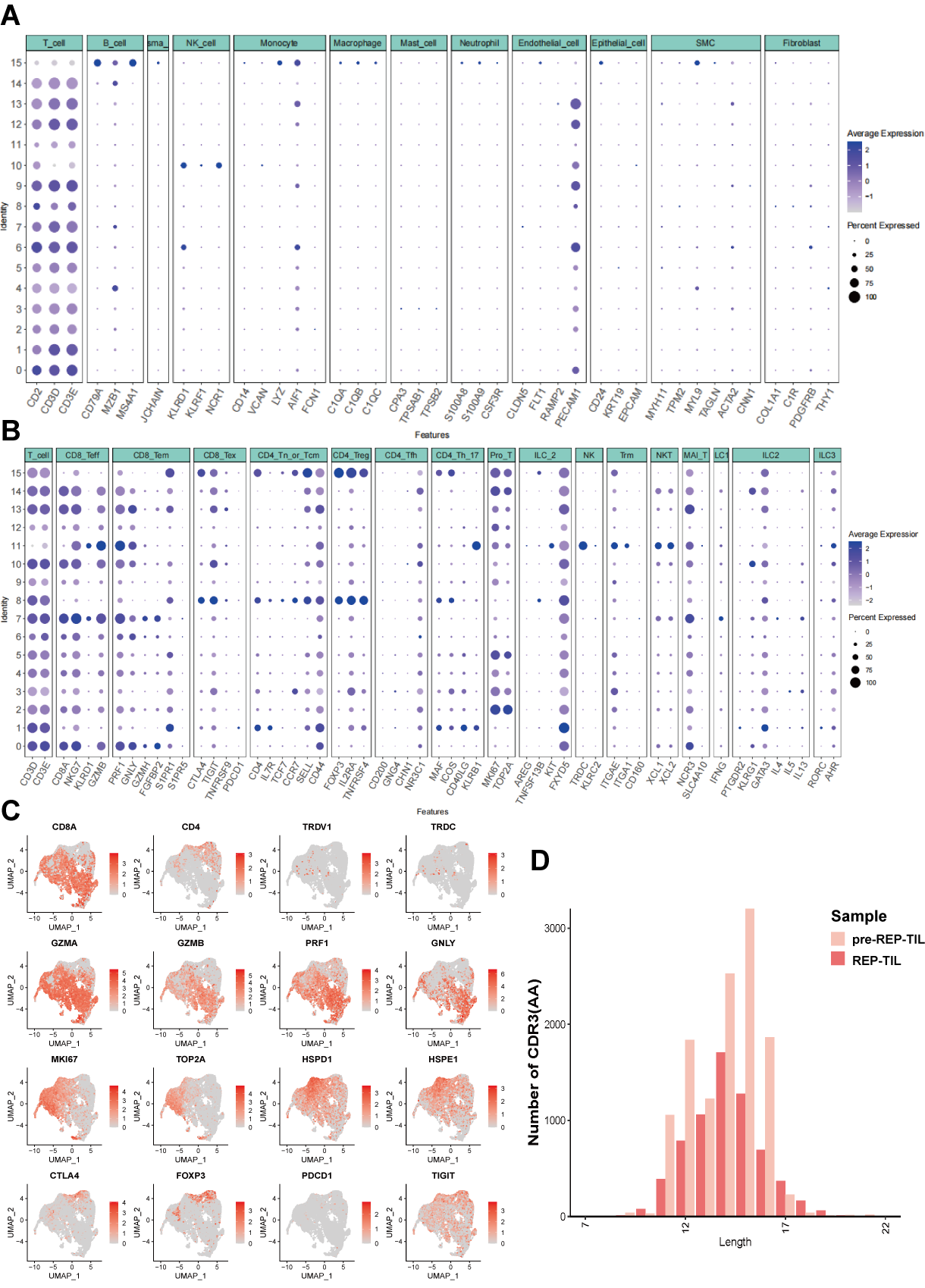


**Supplementary Figure 1. Marker Expression and TCR Distribution Features, related to Figure 2.**

(A-B) Bubble heatmap showing the expression pattern of selected signature genes of major cell lineages (A) and defined T cell subsets (B). Dot size is percentage to the fraction of cells expressing selected genes. Color intensity corresponds to the scaled average expression of selected genes.

(C) Feature plots visualizing scaled expression of representative genes associated with T cell differentiation, cytotoxicity, and immune checkpoints across the T cell UMAP landscape.

(D) Histogram displaying the CDR3 length distribution based on the TCR repertoire.


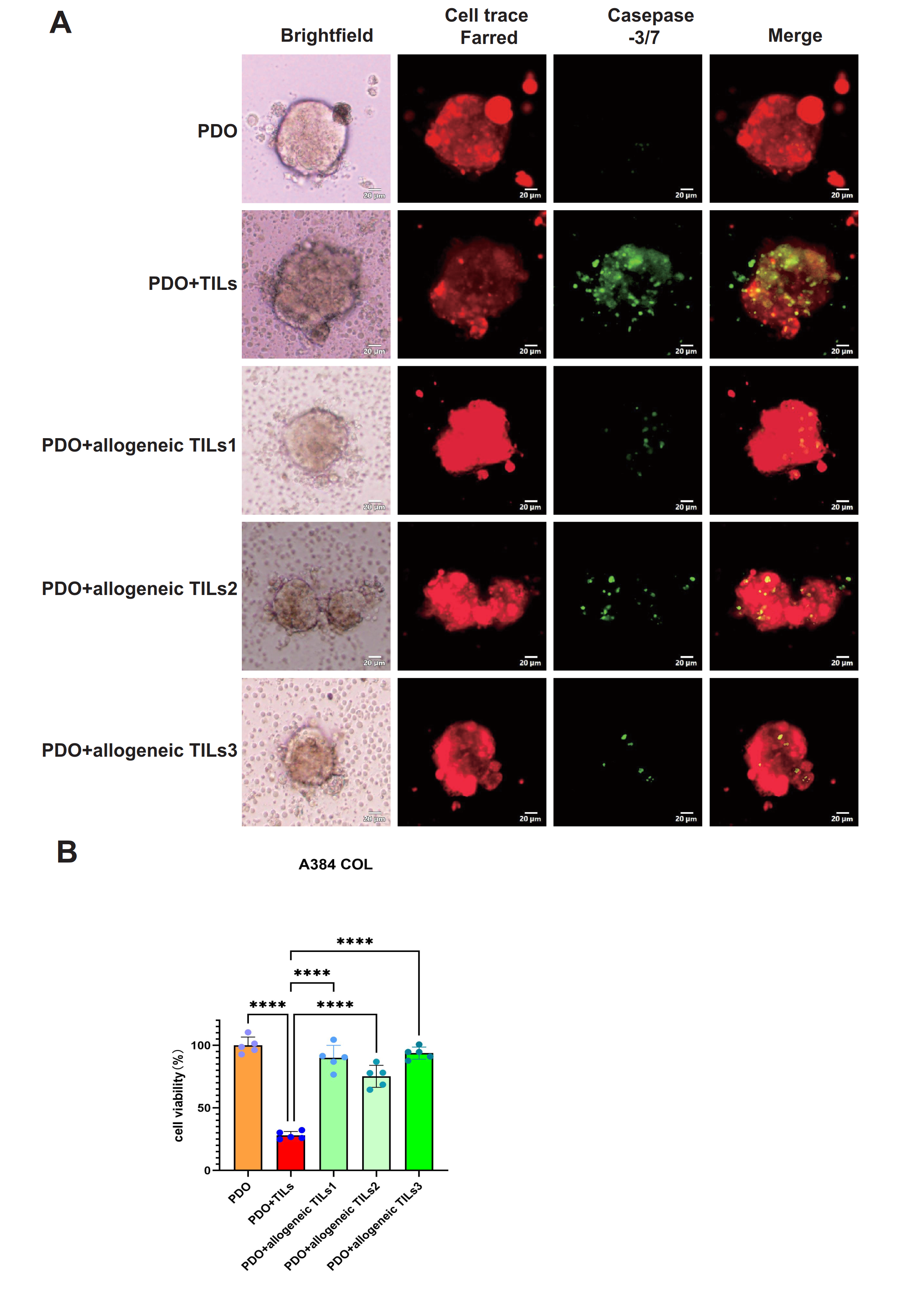


**Supplementary Figure 2. Assessment of Bladder Cancer PDO Susceptibility to autologous/allogeneic TIL-Mediated Killing, related to Figure 4.**

1. Autologous/allogeneic TIL cytotoxicity assay. Representative fluorescence micrographs of CellTrace FarRed-labeled PDO (red; target cells) from Patient A384 after 24-48h co-culture with autologous or allogeneic TIL (allogeneic TILs 1/2/3 from patients A435/A474/A529). Apoptotic cells are identified by activation of a caspase-3/7 sensor (green). Scale bar: 20 µm.
2. Quantitative assessment of TIL-mediated cytotoxicity by ATP-based viability assays. PDO from Patient A384 were co-cultured with autologous TIL or allogeneic TILs from three other patients (A435/A474/A529). Data represents Mean±SD of 5 replicates per group. One-way ANOVA with Tukey’s test. ***P<0.001.
